# Bilateral intertrochanteric fractures in an elderly patient following high-energy trauma: a case report

**DOI:** 10.1186/s12891-024-07432-y

**Published:** 2024-04-22

**Authors:** Zhengjie Tang, Qingshu Wu, Pan Li, Xing Wei

**Affiliations:** 1 Aerospace Center Hospital, Beijing, China; 2 Peking University Health Science Center, Beijing, China

**Keywords:** Bilateral intertrochanteric fractures, Multidisciplinary Team, Elderly, High-energy trauma

## Abstract

**Background:**

Cases of bilateral hip fractures are rare, and even more so are cases of bilateral intertrochanteric fractures. Common causes include trauma, internal diseases, and primary or secondary bone diseases. We report a case of bilateral intertrochanteric fractures in an elderly patient following a severe car accident, a scenario not extensively reported in existing literature.

**Case Presentation:**

We report on an 84-year-old male who suffered severe trauma from a car accident, resulting in multiple injuries and shock state, with pain and limited mobility in both hip joints. After examination and imaging studies, the patient was diagnosed with multiple injuries and bilateral intertrochanteric fractures. Following emergency resuscitation, he was admitted to the orthopedic ward. A pre-surgical multidisciplinary team (MDT) consultation was convened to optimize surgical conditions. The patient underwent successful one-stage bilateral intramedullary nailing. The patient was assisted to stand with a walker on the third day after surgery. Six months post-surgery, the patient resumed outdoor activities.

**Conclusion:**

Managing bilateral intertrochanteric fractures, particularly in the elderly with severe trauma, is notably challenging due to their rarity. However, a coordinated multidisciplinary approach and one-stage bilateral internal fixation can lead to effective treatment outcomes and favorable prognoses.

## Background

Hip fractures are predominantly unilateral occurrences, but bilateral hip fractures represent an extraordinary clinical entity, accounting for a mere 0.3% of all hip fractures [1]. Typically, such fractures in young adults arise from high-energy trauma [2], while in the elderly, they are often a consequence of various internal diseases, such as osteoporosis [3], chronic kidney disease [4], or epilepsy [5].

This rarity is further accentuated in the context of bilateral intertrochanteric fractures. Only a few studies have been reported on bilateral intertrochanteric fractures. Seker A reported the treatment and twelve-year follow-up of a 44-year-old man with simultaneous bilateral intertrochanteric and femoral diaphyseal fractures, along with a proximal tibial fracture, sustained in a motor vehicle accident, emphasizing the effectiveness of single-stage surgical intervention using intramedullary nails and the patient’s ability to return to daily activities with minimal limitations despite long-term mild hip pain [2]. Aydin et al. presents a 76-year-old male patient from Turkey with severe pain and poor mobilization in both hips without a history of major trauma. The patient was treated with closed proximal femoral nailing in both hips in the same operation period. At the last follow-up, 6 months after surgery, the patient was able to walk with aid [6]. Vaishya et al. addresses the rarity of simultaneous bilateral intertrochanteric fractures, usually resulting from severe trauma. It emphasizes the serious nature of these injuries with high morbidity and mortality rates, suggesting early single-stage stable fixation and good rehabilitation as keys to successful management [7].These studies provide valuable insights into the characteristics, management strategies, and outcomes of bilateral intertrochanteric fractures, underlining the importance of a tailored approach to treatment and rehabilitation for these complex injuries.

The case we present, involving an elderly patient who sustained bilateral intertrochanteric fractures following high energy injury (a severe car accident), is not only rare but also unprecedented in medical literature. This case highlights the distinctiveness and the clinical challenges associated with managing bilateral intertrochanteric fractures. The documentation of this case enriches the orthopedic literature by providing a deeper understanding of the etiology, management strategies, and recovery patterns associated with bilateral intertrochanteric fractures in older patients.

### Case presentation

**Fig. 1:**
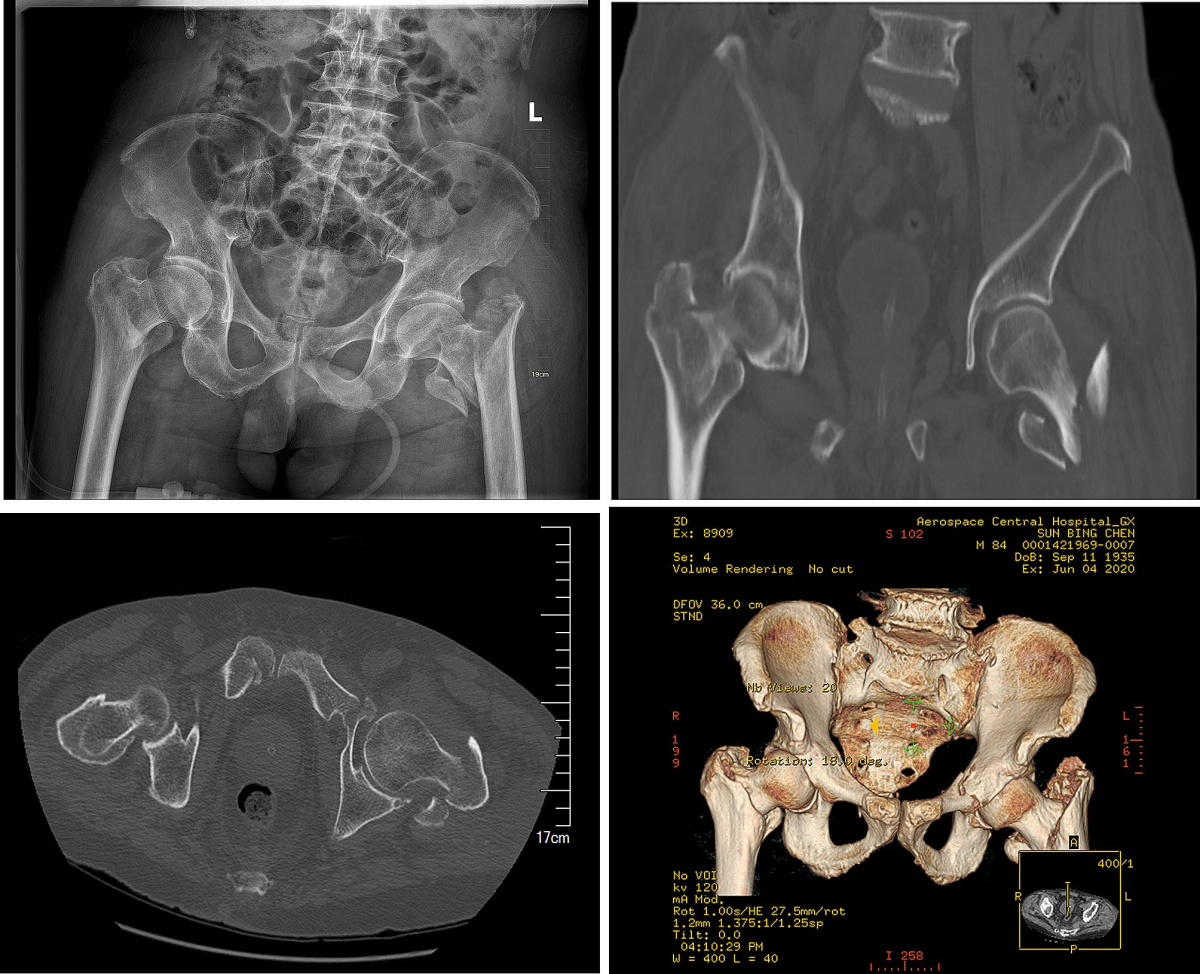
Preoperative imaging of the hip. X-ray and 3D showed bilateral intertrochanteric fractures of the femur, with the left fracture involving the greater and lesser trochanters, which are more obviously displaced, and the right shortened and displaced

An 84-year-old male patient presented to our emergency department with bilateral hip joint pain and limited mobility due to high energy injury (a severe car accident). He suffered from trauma to the hips, chest, and lumbosacral region while riding an electric tricycle that collided with a truck. Physical examination in the emergency room indicated shortening and external rotation deformities in both lower limbs, tenderness in both hip joints (+), and vertical percussed pain of the lower limbs (+). Neurological and vascular examinations of the lower limbs were normal. Radiologic tests confirmed multiple injuries including bilateral intertrochanteric fractures, pelvic fracture, subarachnoid hemorrhage, multiple rib fractures and lung contusion. The bilateral intertrochanteric fractures were obviously displaced (Fig. 1, AO/OTA classification: left AO/OTA 31B2.3, right AO /OTA 31A1.2). Laboratory tests showed a hemoglobin level of 66 g/L, D-dimer of 5719 ug/L (normal 0-500), total bilirubin of 32.6 umol/L (normal 0–26), direct bilirubin of 12.5 umol/L (normal 0–8), and indirect bilirubin of 20.1 umol/L (normal 0–18). Despite no history of prior comorbidities, the patient was admitted to the emergency intensive care unit (ICU)due to his advanced age and poor general condition. The patient received blood transfusions and fluid resuscitation and other symptomatic supportive treatments in the ICU. The vital signs gradually stabilized, but he still complained of pain in his lower limbs (VAS 8). After 6 days, he was transferred to the orthopedic department. A multidisciplinary team (MDT) consultation was convened, involving experts from department of ICU, vascular surgery, cardiology, neurosurgery, hepatobiliary surgery, thoracic surgery, and anesthesiology. The decision was made to perform surgery after administering packed red blood cells and fresh frozen plasma to correct anemia and coagulation abnormalities. Intermuscular vein thromboses were discovered in both lower limbs. However, due to contraindications for anticoagulation therapy because of subarachnoid hemorrhage, an inferior vena cava filter was placed to prevent pulmonary embolism. Symptomatic treatment was provided for obstructive jaundice. The patient’s hemodynamics were stabilized preoperatively, with a hemoglobin level of 96 g/L and nearly normal bilirubin levels. Lactate levels decreased from 1.10 to 0.70mmol/L, and the pH value dropped from 7.454 to 7.382 .

**Fig. 2:**
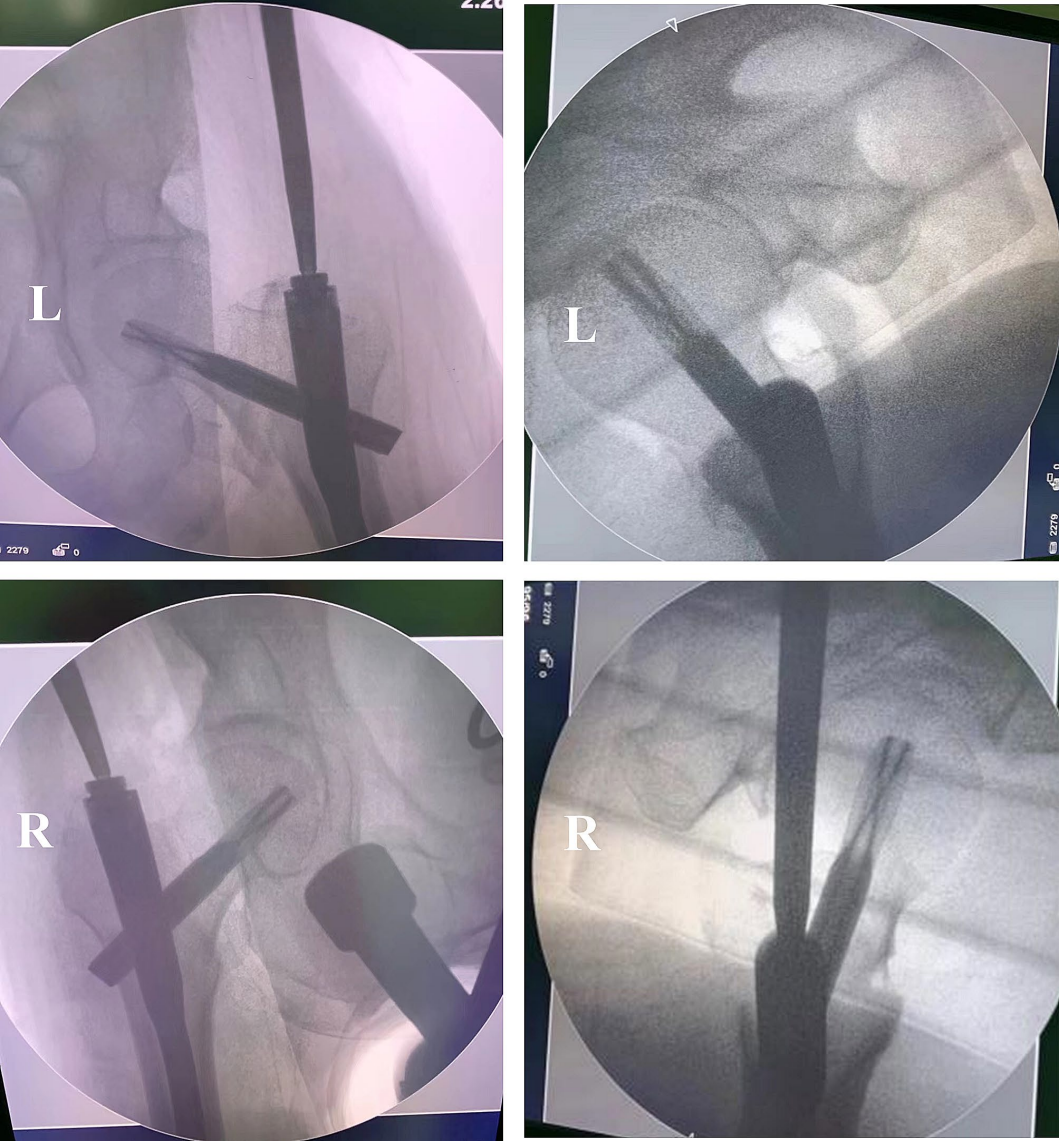
Intraoperative bilateral fixation with PFNA

After 9 days of hospitalization and once the patient’s condition had stabilized, he was scheduled for surgery. The patient underwent a single spinal anesthesia session for bilateral femoral intertrochanteric fracture closed reduction and internal fixation with proximal femoral nail anti-rotation (PFNA). The surgery was smooth (Fig. 2), lasting 2.5 h with a blood loss of 150 ml and no intraoperative transfusion. Postoperative vital signs remained stable, and the patient’s hemoglobin level was 91 g/L on the day of surgery.

**Fig. 3:**
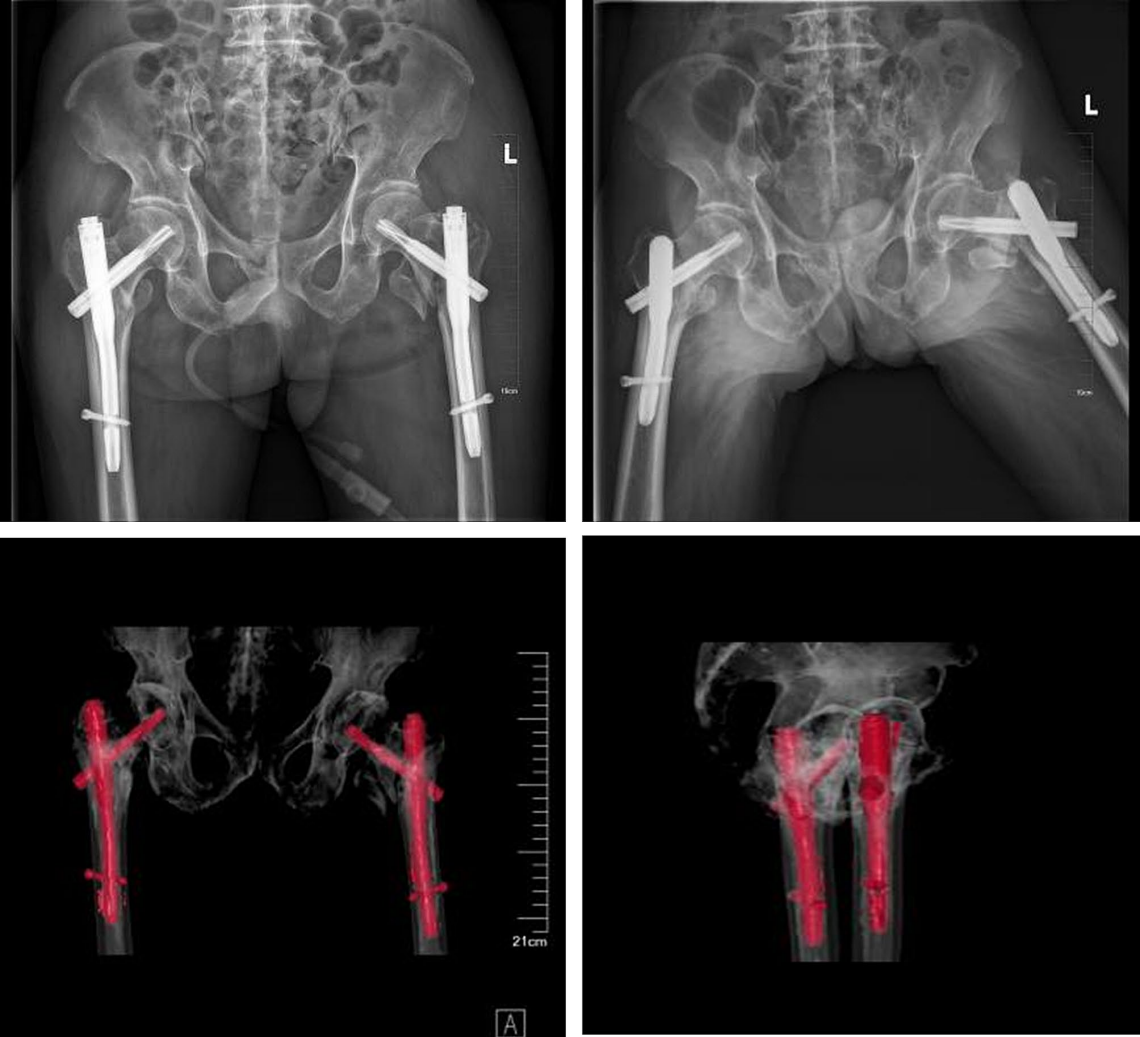
Postoperative X-ray and CT three-dimensional volume rendering (VR) indicated satisfactory position of PFNA without helical blade cuts

Postoperatively, the patient was under cardiac monitoring with normal blood pressure and heart rate. He experienced significant pain relief(VAS 8 to4)and was able to sit up at the bedside. On the third day after surgery, X-ray examination showed satisfactory placement of the internal implants (Fig. 3). The patient began partial weight-bearing ambulation with the assistance of a walker. Due to the COVID-19 pandemic, the patient was discharged one month after the surgery.

**Fig. 4:**
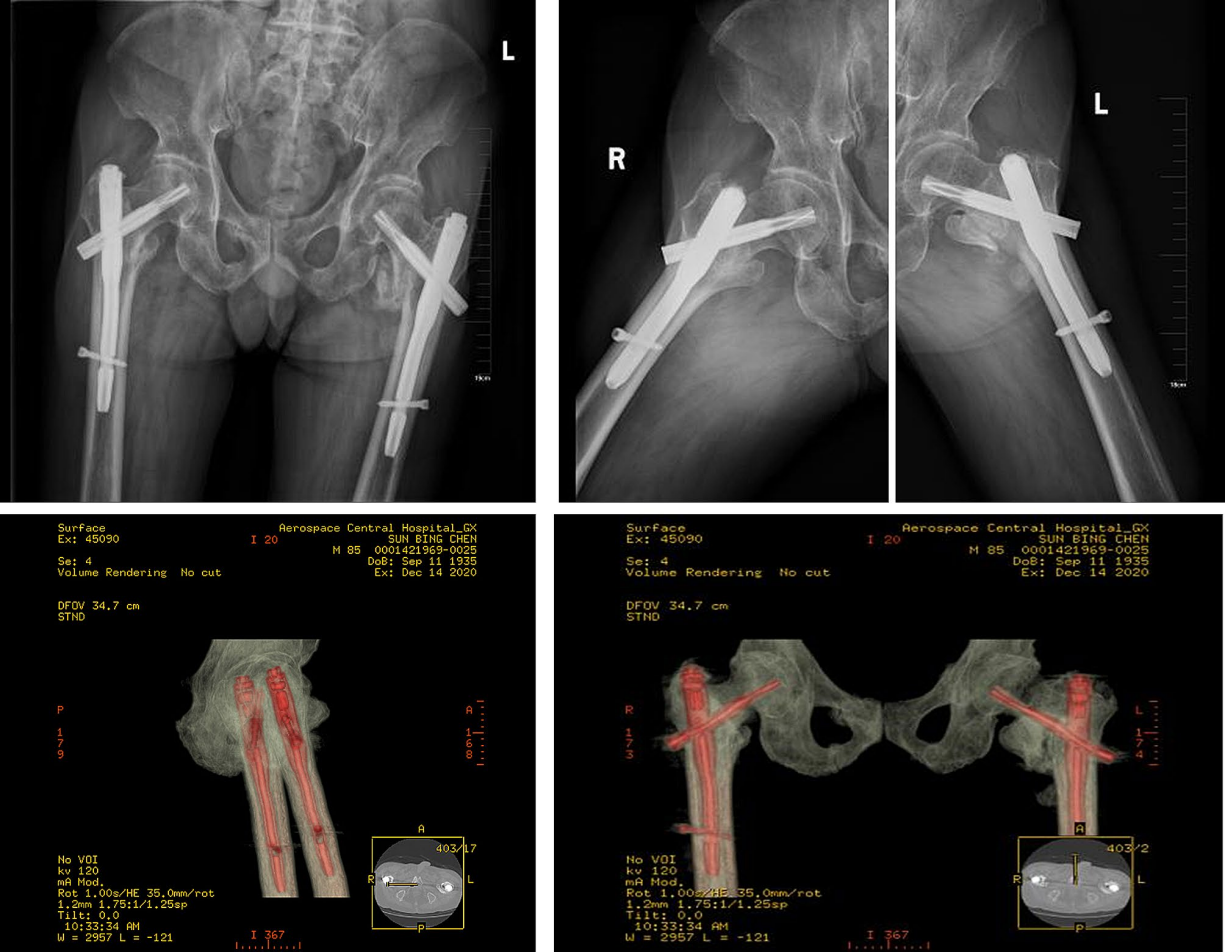
Imaging examination 6 months after surgery. The X-ray and CT showed that the bilateral intertrochanteric fractures of the femur were consistent with healing. The right hip joint was directly healed, with the broken ends of the bones in close contact. There was a large amount of callus formation between the left hip joints, indicating indirect healing

Immediately following the patient’s recovery from anesthesia, a conservative rehabilitation plan was initiated. The rehabilitation program commenced with isometric contractions of the quadriceps muscles and ankle pump exercises to promote circulation and muscle strength. On the 3rd day post-surgery, the patient was encouraged to stand and begin partial weight-bearing ambulation with the assistance of a walker. During the first two weeks post-operatively, the focus remained on bed-based and bedside muscle strengthening exercises, gradually progressing as the patient’s condition allowed. By the 4th week, at the time of discharge, the patient was able to move independently within the ward with the aid of a walker, including performing personal tasks such as using the restroom. Pre-discharge X-rays showed signs of fracture healing. The rehabilitation plan was progressively intensified, and by 3 months post-surgery, the patient had advanced to full weight-bearing indoor activities. At the six-month follow-up, bilateral femoral intertrochanteric fractures had healed (Fig. 4), and the patient was able to walk and perform activities outdoors.

## Discussion

Bilateral hip fractures are rare and associated with poor prognosis. In a study of 2,426 patients with hip fractures, only 8 cases (0.3%) involved bilateral hip fractures, with a mortality rate of 38% [1]. Particularly in patients over 80 years old with low-energy injuries and no additional traumas, the mortality rate was even higher, with deaths occurring within two weeks of hospitalization.

The causes of bilateral hip fractures are complex and often accompanied by massive bleeding, and their treatment requires multidisciplinary team (MDT)consultation. Seker et al. reported a case of a 44-year-old man with bilateral intertrochanteric and femoral shaft fractures from a car accident. Due to early hemodynamic instability and following the principles of damage control orthopedics, the patient underwent early one-stage bilateral surgery once hemodynamic stability was achieved, preventing condition worsening and reducing surgical trauma, thus facilitating recovery [2]. Rajak et al. [8] reported a 41-year-old woman who suffered a hip fracture on one side and an intertrochanteric fracture on the other side due to a fall from standing height. Despite being a low-energy injury, the patient had stage IV chronic kidney disease and severe anemia and was managed with a MDT approach, underwent successful surgery, and was monitored in a high dependency unit for 48 h. Our case involved an elderly patient with high-energy trauma, multiple injuries, and emergency resuscitation, presenting significant treatment challenges. We also adopted a MDT approach to optimize the patient’s pre-surgical condition. The patient was stable during surgery, returned to the general ward postoperatively, and remained stable without the need for specialized treatment.

Previous literature reports that bilateral intertrochanteric fractures are mainly treated with surgery, and there are various surgical options. There are various surgical approaches for treating bilateral intertrochanteric fractures. Rajeev et al. [9]reported a case of a 92-year-old woman with bilateral spontaneous intertrochanteric fractures without a history of trauma. After resuscitation in the emergency department and extensive pre-operative evaluations, she was diagnosed with fractures via MRI and successfully underwent bilateral Dynamic Hip Screw (DHS) fixation. Copuroglu et al [10] described a case of bilateral intertrochanteric fractures caused by a seizure, treated with one-stage bilateral hemiarthroplasty. Aydin [6] reported a case in Turkey of bilateral intertrochanteric fractures treated with proximal femoral nailing, allowing early mobilization, which the patient tolerated well and recovered to their pre-injury state within six months. Intramedullary nailing is an important option for intertrochanteric fractures, enabling early weight-bearing and avoiding complications associated with prolonged bed rest. Considering that osteoporosis in the elderly, we chose proximal femoral nail anti-rotation (PFNA) fixation, which shortened surgery time and allowed for early postoperative mobilization.

Deep venous thrombosis (DVT) is common in hip fractures [11], and the risk is theoretically higher in bilateral cases, although this was not mentioned in previous researches. Our patient had high D-dimer levels preoperatively and developed bilateral intermuscular vein thromboses. Intermuscular vein thrombosis, a type of distal deep venous thrombosis (DVT), has a 6.3% chance of progressing to proximal veins [12]. Hospital-acquired intermuscular vein thrombosis carries a 6% risk of pulmonary embolism (PE) [13]. Surgical manipulation, traction, and postoperative mobilization all increase the risk of thrombus detachment. Since our patient had contraindications for anticoagulant therapy, we opted for preoperative inferior vena cava filter placement to ensure surgical safety and protect the patient during early postoperative activities. Close attention should also be paid to the diagnosis and treatment of venous thromboembolism (VTE) in cases of bilateral hip fractures.

In conclusion, bilateral intertrochanteric fractures resulting from high-energy trauma in elderly patients are extremely rare. Unlike young patients, elderly patients are generally in poor condition and have poorer bone quality. They are usually accompanied by more comorbidities and require MDT consultation to ensure that the general condition is stable before surgery. Multidisciplinary treatment and one-stage bilateral internal fixation can achieve good therapeutic effects and prognosis. The diagnosis and treatment of VTE should not be overlooked in the perioperative period.

## Data Availability

No datasets were generated or analysed during the current study.

## Abbreviations

DVT: Deep venous thrombosis
MDT: Multipledisciplinary Team
PE: Pulmonary embolism
PFNA: Proximal femoral nail anti-rotation
VTE: Venous thromboembolism
